# Behaviour of Acoustically Levitated Drops in Mid-Water

**DOI:** 10.3390/mi14101923

**Published:** 2023-10-10

**Authors:** Jan-Paul Ruiken, Jörn Villwock, Matthias Kraume

**Affiliations:** Department of Chemical and Process Engineering, Technische Universität Berlin, Ackerstraße 76, 13355 Berlin, Germany; ruiken@tu-berlin.de (J.-P.R.); joern.villwock@tu-berlin.de (J.V.)

**Keywords:** acoustic levitation, acoustic streaming, single drop, mass transfer, liquid–liquid systems

## Abstract

A low-impact acoustic levitation system has been developed to study immobilised single drops in liquid–liquid systems. The ability to observe liquid drops several millimetres in diameter for days enables fundamental research into a wide range of mechanisms. Non-invasive optical measurements with excellent optical accessibility are possible. This experimental work provides the basis for mass transfer studies, emphasizing the precise volume determination, signal noise, reproducibility, and the impact of the acoustic field on the drop and its surrounding environment. The setup can be effectively controlled and proves beneficial for research objectives provided that all liquid phases are entirely degassed, and there are no compressible voids present within the liquids. In addition to the precise, uniform, and reliable measurement conditions, we observed no acoustic streaming in the proximity of the drop and there was no significant vibration of the drop. Qualitative observations using rainbow schlieren deflectometry indicate that the nodal or anti-nodal planes of the standing waves can act as barriers to the dispersion of inhomogeneous dissolved substances in the continuous phase.

## 1. Introduction

Acoustic levitation of matter in a gaseous phase is a versatile research tool that has been continuously developed over recent decades. In the design and intended use of the levitation systems, a distinction is made between one- and multi-dimensional approaches.

One-dimensional acoustic levitators in a gaseous environment, where matter is fixed in a specific position, have mainly been used for stationary evaporation [1,2], as micro-reactors [3,4], or for crystallisation experiments [5], to mention some recent ones. The design and construction of these one-dimensional systems is relatively straightforward. However, depending on the application, there may be some obstacles.

Beginning with the availability of affordable ultrasonic transducers, the combination of large numbers of these low-power transducers and their control by FPGAs (field programmable gate arrays) or other micro-controllers opened the door to the multi-dimensional levitation technique. There are many groups using and developing multi-transducer setups for complex matter handling [6,7,8,9,10,11].

With respect to liquids as the continuous phase, matter placement [12,13] and phase separation [14,15] in liquids are areas of research today. Acoustic trapping can be used to handle small particles or droplets without contact and to locate these samples at a specific position. For phase separation, the droplets are a few hundred micrometres in diameter. An overview of the application of ultrasound in phase separation is given in [16], focusing mainly on the petrochemical industry. Most of the investigations are “Lab on a Chip”; they are in the MHz range of acoustic stimulation, working with wavelengths smaller than 1.5 mm with water as the continuous phase. This results in the manipulation of bubbles, droplets, and particles of about Ø 100 µm and smaller [17].

This work aims to highlight the advantages of this unfortunately neglected levitation technique using liquids as the continuous phase with dispersed diameters up to several millimetres. Apfel [18] used acoustic levitation in liquids to measure the strength of liquids in 1971, more than 50 years ago. His developed technique and research were the basis for several publications. In the 1980s, Trinh et al. constructed a simple levitation device to levitate silicone drops in water, with a maximum diameter of 14 mm [19]. The largest mentioned bubble known to us, levitated in water, was reported by Asaki et al., at Ø 12 mm [20]. Experimental conditions, which are important for mass transfer measurements, such as occurring vibrations of motionless drops, the surrounding flow field, and the behaviour of long-term measurements, were not reported or not the focus of these works. Their research focused more on drop oscillation and vibration modes and in situ measurements of interfacial tension and viscosities. This line of research was discontinued in the late 1990s.

We pick up this unique technique and present a system for low-impact liquid–liquid levitation of solvents in water, coupled with exclusively non-invasive optical measurement techniques for real-time investigations with high precision, such as integral mass transfer measurements across the interface and qualitative concentration field visualisation with rainbow schlieren deflectometry. This work describes the developed setup, characterises the levitation environment, quantifies the accuracy, signal noise, reproducibility, and shows the influence of standing waves on the dispersion of inhomogeneous dissolved substances in the continuous phase.

## 2. Experimental Setup

In this section, the developed system is described, limits are named, the measuring procedure is explained, and the image analysis is briefly presented.

### 2.1. Acoustic Levitation System

To acoustically levitate a drop motionlessly and reproducibly for a period from seconds to several days, the acoustic levitation system must be well-adjusted, finely tuned, and controlled in real time to set up a stationary field of standing waves for reliable results.

**Figure 1:**
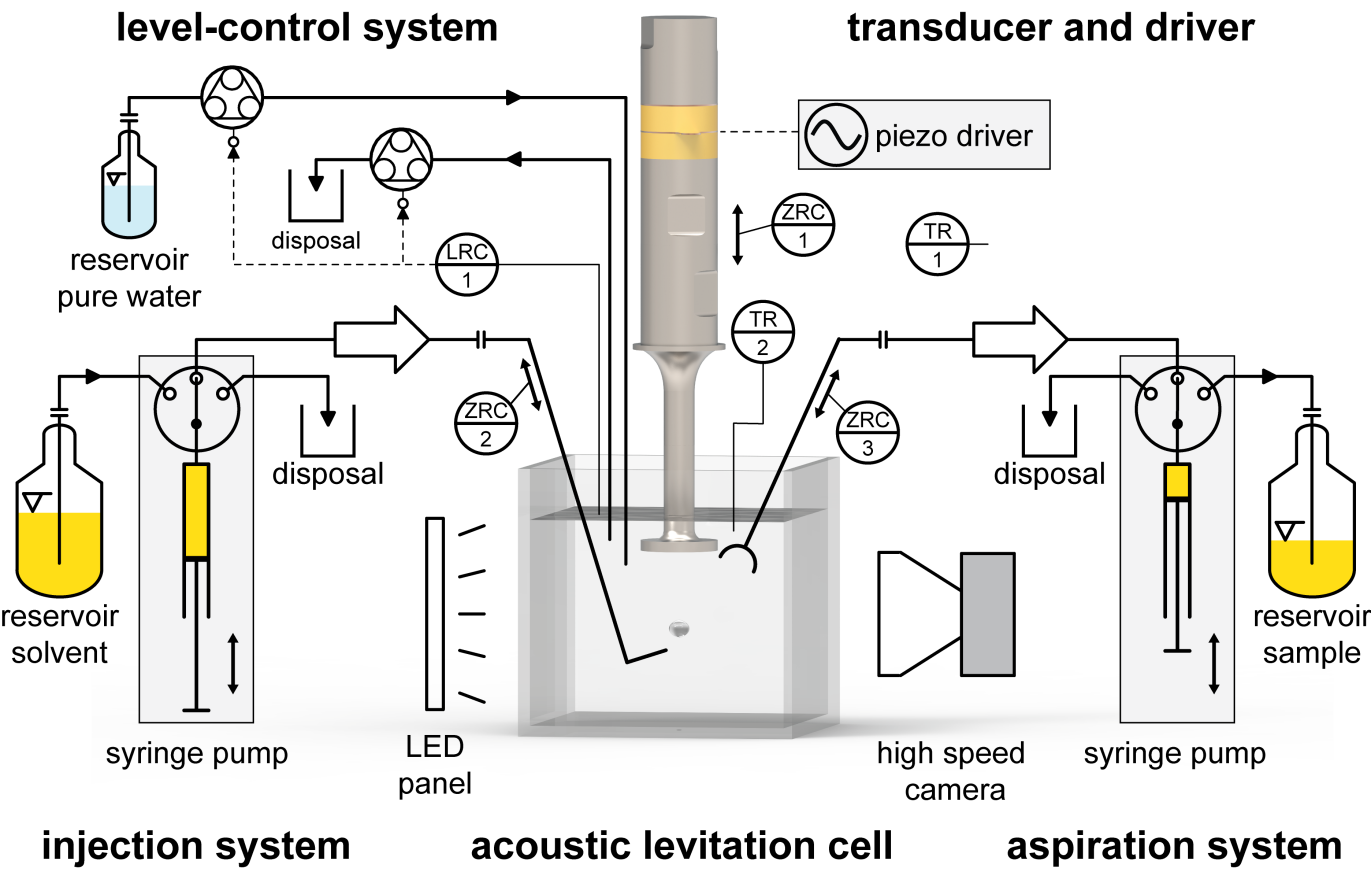
Schematic of the developed acoustic levitation system for liquid–liquid systems.

The schematic of the developed system is shown in Figure 1, consisting of the systems for acoustic levitation, image acquisition, level control, injection, and aspiration.

The single-axis levitator consists mainly of a custom-made well-matched submerged plate-tip Langevin transducer. The transducer body, the two PZT4 ceramics, and the plate-tip are Ø 15 mm in size, and the total length is 125 mm. The horn is made of Ti-6Al-4V and tapers from Ø 15 to 6 mm to amplify the horn motion. Levitation is achieved at the resonant frequency $f_{r}$ of approximately 66.5 kHz when the horn is immersed in water to a depth of approx. 13 mm. An optical 125 mL cubical cuvette (Series 704, Hellma GmbH & Co. KG, Mullheim, Germany) contains the ultrapure water. The transducer is driven by an ultrasonic amplifier (PDUS210-V4, PiezoDrive, Shortland, Australia), with capabilities of real-time phase tracking and impedance detection. The outer bottom of the cuvette acts as a reflector to create the necessary standing wave for acoustic levitation. The distance from the tip of the transducer to the bottom of the cuvette is controlled by a motorised linear stage (MTS50-Z8, Thorlabs Inc., Newton, NJ, USA). A precise and constant water level is crucial for good levitation results; it is set by two peristaltic pumps (T100-S301/WX10-14-H, Longer Precision Pump Co., Ltd., Baoding, China) and controlled with the signal of an ultrasonic distance sensor (pico+15, microsonic GmbH), resulting in a 0.1 mm level control accuracy. One peristaltic pump is used to fill and one to drain and flush the system between measurements. The temperature of the continuous phase and the ambient temperature are monitored by two Pt100 temperature sensors (Ø 2 mm, 4-wire, Class B, TC LTD., Uxbridge, UK).

The shape and location of the drop can be observed using a monochrome high-speed camera (CamRecord CL600x2, Optronis GmbH, Kehl, Germany), coupled with a telecentric lens (S5LPJ0422 Correctal TL/2.0, Sill Optics GmbH & Co. KG, Wendelstein, Germany), a frame grabber (microEnable IV AD4-CL, Silicon Software GmbH, Mannheim, Germany), and an LED panel (TH-100x100RD, CSS Inc., Kyoto, Japan) for transmitted illumination. The established field of view is 7.68 mm × 7.68 mm.

The automated drop injection and aspiration system is the main feature of this setup. The solvent drop is injected just below the only pressure anti-node with a hook-shaped glass capillary guided by a fast-moving linear actuator (L12-20PT-6, IR ROBOT CO., Ltd., Bucheon, Korea). After injecting a defined volume, the linear actor retracts the capillary. The observation of the drop starts. After a chosen contact time, a micro glass funnel (custom design, WJM Glas GmbH, Berlin, Germany) is positioned over the levitating drop by a slow-moving linear actuator (L12-80PT-6, IR ROBOT CO., Ltd., Bucheon, Korea). The ultrasonic field forces the funnel into resonance and creates its own acoustic field; the drop is drawn into the funnel. Both injection and aspiration tools are connected to syringe pumps (PSD/3-mini, Hamilton Company, Bonaduz, Switzerland) for precise liquid handling.

To suppress external vibrations and to ensure vibration-free measurements, the complete system is mounted on a portable optical breadboard and then placed on an air-damped optical table (CleanTop series 783 with Micro-g pneumatic legs, TMC). For external shock detection, an acceleration sensor (QG40N-KAXYZ-1.5, DIS Sensors, Witten, Germany) and an inclination sensor (QG40N-KDXYh-030, DIS Sensors, Witten, Germany) are placed on the optical breadboard. All components of the setup communicate via a modular data acquisition system (cRIO 9074, National Instruments, Austin, TX, USA) equipped with measuring cards and controlled and operated with LabVIEW (Version 2021, National Instruments, Austin, TX, USA).

The second accessible optical axis is used for qualitative 2D concentration measurements with a rainbow schlieren deflectometry system (RSD) developed by Schulz et al. [21] and further improved by Junne et al. [22].

#### 2.1.1. Specifications

The setup is designed and optimised for one operating point of levitation, to ensure the best levitation results in terms of low vibrations, high reproducibility, and robustness. Within an ambient temperature range of 21 ${}^{\circ }$C ± 3 K and ultrapure water as the continuous phase, the field of standing waves can be set up easily. The system is designed to levitate in the only anti-node, which is located in the centre of the cuvette. A variety of solvents with a negative acoustic contrast factor ϕ<0 can be levitated in this system as the dispersed phase. The acoustic contrast factor ϕ was defined by Gor’kov [23] as follows:

(1) $$\phi =\frac{\rho_{d}+\frac{2}{3}\left(\rho_{d}-\rho_{c}\right)}{2\cdot \rho_{d}+\rho_{c}}-\frac{1}{3}\cdot \frac{\rho_{c}\cdot c_{c}^{2}}{\rho_{d}\cdot c_{d}^{2}},$$

with the densities ρ and the speed of sound *c* of the dispersed (*d*) and continuous phase (*c*).

The minimal initial drop diameter is determined by the detachment behaviour of the injection capillary. If a drop is levitating, there is no lower diameter limit. The largest injected drop that can be levitated and gives good levitation results is Ø 5.5 mm. Larger drops require a higher power input to stay in position, which leads to cavitation and resulting vibrations. The upper limit in this system is a deformed and slightly vibrating Ø 10 mm drop.

The dispersed and continuous phases must be completely degassed. Otherwise, the field of standing waves would immediately cause cavitation, resulting in vibrations and acoustic streaming. Table 1 summarises the specifications of the developed system.

#### 2.1.2. Measuring Procedure

The plate-tip of the Langevin transducer is positioned at a specific height and the amplifier is enabled with activated phase tracking, resulting in a resonant frequency of $f_{r}\approx$ 66.5 kHz, a sinusoidal driving voltage of $V_{pp}=$ 30 V, a phase shift of θ=−10°, and a maximum power input to the transducer of $P_{t}\approx$ 1 W. The bottom of the cuvette acts as a reflector. The open cuvette is automatically filled with ultrapure water to a preset level. To levitate a drop with ϕ<0, the power input of the transducer is between 0.7 and 1.0 W. The impedance |Z| of the transducer, or rather the resonating system, is a good indicator that the system is able to levitate and is set up correctly. To achieve a levitation result with low vibrations, there should be no gas bubbles in the cuvette or gas dissolved in the phases. Any compressible voids or materials immersed in the continuous phase will cause vibrations and acoustic streaming. Therefore, only glass and titanium grade 5 are placed inside the cuvette, and even PTFE should be avoided.

The injection system is flushed and filled, and then the injection capillary is attached to the linear actuator. The first drops must be injected in the manual control environment, before starting the automated measurements.

A measurement begins with the level control system adjusting the water level in the cuvette. Before a drop of a certain volume is injected, the high-speed camera (HSC) is activated and acquires images continuously at 10 Hz. The linear actuator moves into the cuvette and injects the drop near the anti-node, and then exits the cuvette. The contact time can be selected according to the type of experiment. At the end of a measurement, the drop is aspirated by a glass funnel that moves above the drop. When the ultrasound is turned off, the drop enters the funnel and is aspirated. This procedure can be repeated according to the desired design of experiments.

### 2.2. Image Analysis

The images are evaluated with MATLAB (R2022b, MathWorks), and the main workflow for image processing is illustrated by a sample code in Appendix A.1.

The resting levitating drops are rotationally symmetric about the z-axis; therefore, 2D images are used to calculate the drop volume. The images contain location and different shape properties of interest. With an image resolution of (1024 px)², a spatial image resolution of 7.5 µm/pixel, and sharp drop outlines, precise calculations are possible.

**Figure 2:**
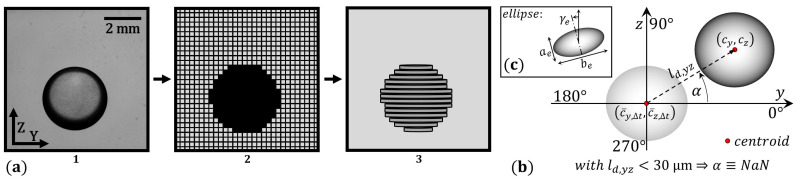
(**a**) Volumetric measurements, image to volume: (1) acquired image, (2) binarised image, and (3) rotated pixel layers forming the disks. (**b**,**c**) Visualisation of the introduced measures.

The drop volume $V_{drop}$ is acquired by rotating every pixel line of the binarised 2D image around the z-axis and summing up the volume of every disc of 1 px height (Figure 2a).

The volume-equivalent diameter $d_{s}$ of a perfect sphere is defined as

(2) $$d_{s}={\left(\frac{6\cdot V_{drop}}{\pi }\right)}^{1/3},$$

and will be further referred to as the diameter *d* of a drop.

A drop’s covered distance $l_{c}$ can be determined by calculating the cumulative sum of each centroid shift from frame to frame, which gives a good overview of a measurement.

With changing system parameters, such as the drop’s volume, water level, temperature, etc., the resonant system is slowly affected, and the drop’s position changes accordingly. These changes are rather slow compared to spontaneous vibrations of other nature. For analyzing the movement, the centroid $c_{y/z}$ will further describe the position of the drop. To detect and quantify these faster movements, the centred moving average ${\bar{c}}_{y/z,\Delta t}$ (CMA) of the centroid $c_{y/z}$ over a time interval Δt [−*k*, *k*] is calculated regarding the y-axis and z-axis:

(3) $${\bar{c}}_{y,\Delta t}=\frac{1}{2k+1}\sum_{j=-k}^{k}c_{y,t(j)}\;\mathrm{and}\;{\bar{c}}_{z,\Delta t}=\frac{1}{2k+1}\sum_{j=-k}^{k}c_{z,t(j)}.$$

A schematic description of a drop and characteristic measures are visualised in Figure 2b. The individual y- and z-deflection lengths $l_{d,y/z}$ between the current centroid $c_{y/z}$ and the CMA centroid ${\bar{c}}_{y/z,\Delta t}$ are calculated as follows:

(4) $$l_{d,y}=c_{y}-{\bar{c}}_{y,\Delta t}\;\mathrm{and}\;l_{d,z}=c_{z}-{\bar{c}}_{z,\Delta t}.$$

The overall deflection length $l_{d,yz}$ is then acquired through Pythagoras:

(5) $$l_{d,yz}=\sqrt{{\left(l_{d,y}\right)}^{2}+{\left(l_{d,z}\right)}^{2}}.$$

The angle of deflection $\alpha_{deg,y\to }$ can be calculated as follows:

(6) $$\alpha_{deg,y\to }=\frac{180}{\pi }\cdot arccos\left(\frac{c_{y}-{\bar{c}}_{y,\Delta t}}{l_{d,yz}}\right),$$

and will only be defined when the corresponding deflection length $l_{d,yz}$ is at least four pixels of the high-speed camera (HSC) image. Deflections under 30 μm in length are not considered to be significant movements.

The idle velocity $v_{i}$ represents the drop’s 2D moving speed. It is obtained by dividing the covered distance between every frame by the elapsed time of the time interval Δ*t* [$t_{0}$, *t*]:

(7) $$v_{i}=\frac{\sum_{j=2}^{m}\sqrt{{\left(c_{y,j-1}-c_{y,j}\right)}^{2}+{\left(c_{z,j-1}-c_{z,j}\right)}^{2}}}{\Delta t}.$$

The frame rate at which the images are captured greatly affects this measure.

The almost spherical shape of the levitated drops is characterised by the parameters $a_{e}$, $b_{e}$, $\gamma_{e}$ of a fitted ellipse (Figure 2c). The roundness is described by the circularity *C*:

(8) $$C=\frac{4\cdot \pi \cdot A_{p}}{p^{2}},$$

with the area $A_{p}$ and the perimeter *p* of the 2D image of the drop.

## 3. Materials and Experiments

Ultrapure water is always used as the continuous phase in this work. Toluene is the main solvent. The acoustic contrast factor in water is ϕ<0 and, therefore, can be levitated in the developed system. For rainbow schlieren deflectometry measurements of the concentration streaks, three additional solvents are introduced. For visualisation over the levitating drop, butyl acetate and 1-butanol ($\rho_{d}<\rho_{c}$, ϕ<0) with a solubility of 6.8 and 84 g/L are used. To visualise the flow below the drop, propylene carbonate ($\rho_{d}>\rho_{c}$, ϕ>0) with a solubility of 240 g/L was chosen, but cannot be levitated in the only anti-node as a pure substance. By mixing 5vol% propylene carbonate with toluene, the mixture of solvents can be levitated (ϕ<0) and propylene carbonate will be visible as the main transfer component (TC) by rainbow schlieren deflectometry. The properties of the pure substances and the mixture are listed in Table 2.

### 3.1. Evaluation of the Precision of Image Analysis

The absolute accuracy and signal noise of the measured volume and resulting volume-equivalent diameter of a perfect sphere are determined by using optical calibration targets, with precise chrome dots imprinted on glass. These dots represent drops of Ø 1.00 and 5.00 mm. The targets are captured for three minutes at a frame rate of 10 Hz.

### 3.2. Behaviour of Levitating Drops

In order to examine the behaviour of levitating drops from injection to long-term investigations, three different time intervals are analysed:

- 0–20 s, representing the injection behaviour;
- 1–2 min, representing the short-term behaviour;
- 1 min–8 h, representing the long-term behaviour.

t = 0 s is the moment of drop detachment, as the injection capillary gets pulled out. The measurements focus on motion, shape, injection accuracy, signal noise, and reproducibility. The system toluene/water is used for these investigations, because of its low aqueous solubility and, therefore, small volume change during a measurement. The initial diameters for the experiments are in the range of Ø 3–5.5 mm.

**Injection behaviour (0–20 s)**: The dispersed phase is injected and placed close to the pressure anti-node forming a motionless drop attached to the capillary. The capillary is pulled out and the initial drop detaches and moves towards the pressure anti-node and oscillates around its final position for a short period of time. The focus is on the covered distance, the decay of the movement, and the decay of shape oscillations. Starting with the detachment from the capillary, the drop is observed over 20 s at a frame rate of 500 Hz. This experiment represents a standard injection procedure.

**Short-term behaviour (1–2 min)**: The injection movement has completely decayed after one minute, representing the system at rest. These measurements are made to quantify the occurring vibrations (deflection length $l_{d,yz}$), the accuracy of the injected volume, including the signal noise, idle velocity, and other shape measures. The short-term behaviour of one minute is observed at a frame rate of 10 Hz.

**Long-term behaviour (1 min–8 h)**: The injection movement has completely decayed after one minute. The long-term behaviour of drops with varying initial diameters is observed over 8 h at a frame rate of 10 Hz to quantify the occurring vibrations (deflection length $l_{d,yz}$) and the reproducibility of the measurements. Under ambient conditions in the lab, the evaporation speed of water in the open cuvette is about 0.1 mm/h. The water needs to be refilled to the initial level every four hours. The impact on the deflection length by refilling the cuvette is shown below.

### 3.3. Flow Field in the Vicinity of the Drop

Particle image velocimetry and rainbow schlieren deflectometry measurements are carried out to quantify acoustic streaming and the influence of standing waves on the spread of inhomogeneous dissolved substances in the continuous phase.

**Particle Image Velocimetry (PIV)**: For quantitative acoustic streaming measurements, the continuous phase is initially homogeneously seeded with Rhodamine B particles (Ø 20–50 µm). A Ø 4 mm toluene drop is then injected and PIV measurements are conducted.

**Rainbow Schlieren Deflectometry (RSD)**: For non-invasive partial acoustic streaming and flow field measurements in the continuous phase, RSD measurements for qualitative visualisation are performed. The impact of the standing waves on the propagation of solvents in the continuous phase is also qualitatively investigated with this method.

RSD visualises gradients in the refractive index (RI) of a system and, therefore, the concentration field in the system, if the RI values of the dispersed and continuous phases differ. The systems with significant aqueous solubilities, butyl acetate/water, 1-butanol/water, and toluene/water/propylene carbonate, are investigated. The drops are injected and the resulting concentration field around the drop is visualised by the RSD system developed by Schulz et al. [21] and further improved by Junne et al. [22]. A radial filter is used for the measurements in this work. Two of the three solvents have a lower and one has a higher density compared to the continuous phase, resulting in different vertical main directions of mass transfer.

## 4. Results and Discussion

Firstly, the accuracy of the volume determination is analysed, which is crucial for the application of this technique. Then the behaviour of the drops from injection to long-term measurements is examined. The importance of liquid phase degassing in this technique is discussed. Finally, the influence of the standing waves on the drop’s vicinity with different dispersed substances is examined.

### 4.1. Evaluation of the Precision of Image Analysis

The absolute accuracy of the optically acquired volume by measuring the optical dot calibration targets over three minutes is shown in Table 3, giving the acquired volume with the calculated diameter (Equation (2)) and the resulting deviation. The spatial resolution of 7.5 µm/pixel has been previously calibrated and is independent of these measurements.

This results in a maximum error of the optical acquisition of 0.1581% in diameter and 0.4735% in volume, which occurs at the smallest diameter. Overall, the volume error can be effectively approximated to ±0.5% for diameters greater than 1 mm when the drop is at rest and does not change its shape. The telecentric objective has low distortion, so the acquired volume is independent of position. The signal noise of the volume over 180 s of a fixed Ø 1.00 mm target is 2.01‰ and decreases with size to 0.28‰ for a Ø 5.00 mm target. The noise in the image results in an idle velocity $v_{i}$ (Equation (7)) of less than 60 µm/min at a frame rate of 10 Hz and is, therefore, negligible.

### 4.2. Behaviour of Levitating Drops

The basis for accurate mass transfer measurements of immobilised drops is certainly the stability in the position of the levitated drop and a uniform shape during the contact time itself. Every movement in position or shape oscillations results in increased mass transfer. We take a close look at several time periods to map a complete long-term measurement. The focus is on the shift of the centroid and its circularity in addition to the optically acquired volume. Within this subsection, the toluene/water system is examined due to its low aqueous solubility and, therefore, slow volume change within a measurement.

**Injection behaviour (0–20 s)**: After the volume of the drop is injected, the drop is still attached to the capillary. When the capillary is pulled back, the drop detaches and moves towards the pressure anti-node and orbits around it for a short time. An injection of a Ø 3.5 mm drop is shown in Video S1 of the Supplementary Material.

**Figure 3:**
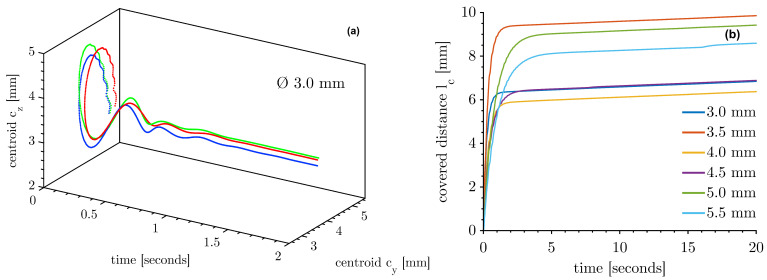
Movement of drops after injection. (**a**) Path of the centroid *c* in y- and z-direction for three Ø 3 mm drops. (**b**) The covered distance in the yz-direction of drops with different initial diameters.

Figure 3a shows representative two-dimensional paths of three Ø 3 mm drops from the point of detaching and the following two seconds approaching the resting position. The first movement upwards is caused by the buoyancy of the drop which then deflects toward the pressure anti-node and orbits around it until the momentum decays. Drops of different diameters behave similarly. This behaviour is analysed in more detail below.

Figure 3b shows the trajectories of drops with differing initial diameters. The initial covered distance does not correlate with the diameter and is in the range between 5 and 10 mm. Movement in the x-direction is not captured with this setup; the actual covered distance is certainly higher. A three-dimensional movement trajectory would lead to a smaller variation in the initial range of the covered distance. After the decay of the initial motion, the curves of the drops in Figure 3b are parallel and have a slope of ~27 µm/s or ~1.62 mm/min, respectively. This slope can be understood as the velocity of the centroid in the yz-direction and is the idle velocity $v_{i}$ (Equation (7)). The idle velocity characterises the measurement and how the standing waves affect the vibrations of the drop. It is a simple and reliable indicator of a successful and usable measurement. After injection, a jump in the average idle velocity can indicate a deviation in levitation (e.g., Figure 3b, Ø 5.5 mm drop at 16.5 s). Considering the x-direction, we estimate that the three-dimensional movement is below 40 µm/s and 2.4 mm/min, respectively. The injection behaviour measurements are made at a frame rate of 500 Hz, so the signal noise of the centroid increases the idle velocity compared to the 10 Hz measurements below.

**Figure 4:**
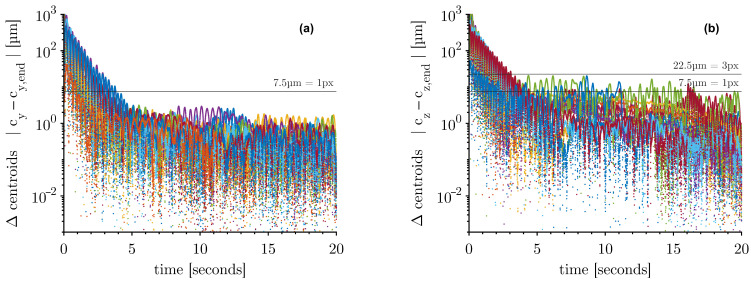
The absolute value of the deviation between the actual centroid and the centroid at 20 s for all measurements (Ø 3–5.5 mm) differently coloured: (**a**) with respect to the horizontal axis y and (**b**) with respect to the vertical axis z.

A more detailed insight into the decaying behaviour is given in Figure 4. The trajectories of the distance between the actual centroid and the centroid at 20 s of the horizontal and the vertical axis of all measurements with different diameters are shown.

After four seconds, the horizontal deflection (Figure 4a) falls below the edge length of one pixel of the HSC image (7.5 µm). For the vertical deflection, the initial motions decay more slowly (Figure 4b). Most drops decay within the first three seconds below 7.5 µm. A minority of the drops need a longer decay time, some taking up to 16 s. The drops are, in conclusion, about to be almost stationary in position after 16 s at the latest. The drops are more stable horizontally than in the vertical direction.

**Figure 5:**
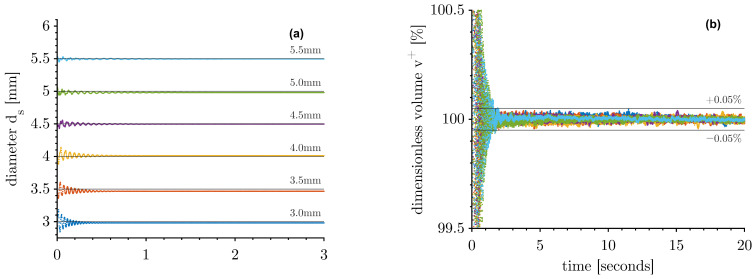
Injection behaviour of the volume of different diameters: (**a**) the acquired volume within the first three seconds after detachment and (**b**) dimensionless volume $v^{+}=v_{t}/v_{t=20s}$.

The acquired volume of these initially moving and deformed drops is shown in Figure 5a, which illustrates one representative drop of each diameter. When the oscillation in shape decays, the calculated volume is constant and results in the actual volume of the drop. At this point, the drops show a circularity *C* of more than 0.98, and the drops can be assumed to be rotationally symmetric about the z-axis. With increasing drop size, the time of oscillation lasts longer, and this behaviour can also be seen in Figure 3b by reaching the constant slope more slowly. The variation of the dimensionless volume $v^{+}$ (recorded volume/recorded end volume) is less than ±0.05% (Figure 5b) two seconds after injection.

In conclusion, the decay of the shape is faster than the decay of the position. The acquired drop volume is, therefore, reliable after two seconds. A total of 20 s after the detachment from the capillary, the movement is stable. In addition, the transient circularity *C* of an acquired drop can be used to verify whether the drop volume is reliable. To acquire volumes of asymmetrical shape caused by dynamic drop movements, at least one additional optical axis is necessary. This additional axis would also allow the measurement of the three-dimensional idle velocity of a drop.

**Short-term behaviour (1–2 min)**: One minute after the drop is injected, the initial movement of the drop has dissipated. For this time interval, about 600 images were analysed for drops of different initial diameters. The characteristic values for one representative drop of every diameter are summarised in Table 4.

The optical volume acquisition has a signal noise of less than 0.5‰ in the range of Ø 3–5.5 mm drops, almost independent of the diameter. The signal noise in the volume of a Ø 5 mm drop is 0.27‰ and the raw optical signal noise of a Ø 5.00 mm optical target is 0.28‰ (Table 3). The signal noise in volume is, therefore, due to the optical acquisition and does not originate from any induced vibrations. The absolute deviation in the injected volume of the drops is caused by the random uncertainty of the injection system. By withdrawing the injection capillary, some of the dispersed phase may be left on the capillary, lowering the injected volume. The interfacial tension also affects the deviation of the injection of the target volume. Since this uncertainty is quantifiable, it has no negative effect on the measurements. The acquired deflection length $l_{d,yz}$ is in the sub-pixel range; the drops are overall not moving or vibrating significantly. For all drops, the two-dimensional idle velocity at a frame rate of 10 Hz is less than 0.55 mm/min. In addition, taking into account the undetected movement in the x-direction, the three-dimensional idle velocity caused by the occurring vibrations can be estimated to be less than 0.8 mm/min.

In terms of shape, the drops are of a spherical nature with a minimum circularity *C* of 0.99 (Table 4). The larger the diameter, the more it deviates from a perfect sphere. The cubic cuvette and the transducer are not exactly concentric; therefore, there is a small negative offset in the tilt $\gamma_{e}$.

**Figure 6:**
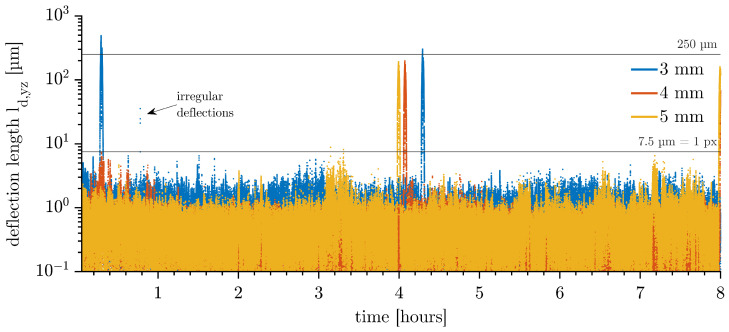
Deflection length $l_{d,yz}$ with Δt = 120 s of toluene drops, 8 h long-term behaviour. The spikes occurring at 0.3, 4.0, 4.1, 4.3, and 8.0 h indicate a change in position caused by the need to relevel the water every four hours.

**Long-term behaviour (1 min–8 h)**: Figure 6 shows the deflection length $l_{d,yz}$ of a Ø 3, 4, and 5 mm drop. The irregular peaks at four-hour intervals are caused by the deliberate slow relevelling of the water in the cuvette. A changing water level affects the position of the drop mainly in the z-direction. Apart from these outliers, the deflections of the drops are almost all below 7.5 µm and, therefore, extraordinarily stable in position, regardless of the drop diameter. A time-lapse of a long-term measurement of a Ø 3 mm drop is shown in Video S2 of the Supplementary Material. External vibrations cannot be completely ruled out, as can be seen by three dots above 7.5 µm in Figure 6 of the Ø 3 mm drop at 0.8 h.

**Figure 7:**
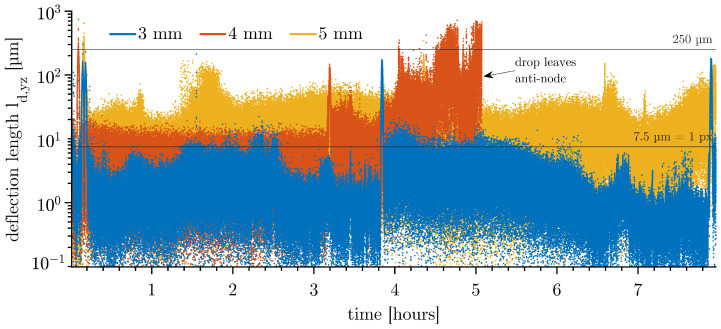
Deflection length $l_{d,yz}$ with Δt = 120 s of toluene drops of non-degassed systems, 8 h long-term behaviour. At 5.1 h, the Ø 4 mm drop left the anti-node; gas bubbles formed and caused the levitation to fail.

The drops vibrate strongly when the dispersed and continuous phases are initially saturated with air. This is shown in Figure 7 for three example drops of different diameters. The trajectories do not show a stable behaviour. Overall, the measurements are not reproducible. The ultrasonic field degasses the phases, resulting in many small gas bubbles on the walls of the cuvette. These compressible cavities absorb the ultrasonic waves and strongly influence the resonant system. This can lead to a failure of the drop levitation, as seen for the Ø 4 mm drop leaving the anti-node at 5.1 h with no further signal acquired.

The idle velocities from 1 minute to 8 h, including the deflections caused by relevelling the water, are given in Table 5 for the previously shown measurements of degassed (Figure 6) and non-degassed (Figure 7) systems.

The measurements show that the standing waves do not induce significant drop vibrations in systems that have been degassed. It can be estimated that up to 10% of the idle velocity of the degassed systems is caused by the signal noise of the optical acquisition system (cf. Table 3, Ø 5 mm). The idle velocities of the long-term measurements are generally lower than the short-term measurements. We believe that the continuous exposure to the acoustic field degasses the system even more. This results in fewer vibrations over time and also prevents the dissolution of ambient air components into the continuous phase in the open cuvette. To achieve a high level of vibrations and idle velocity, the dispersed and continuous phase should not be degassed or can be additionally gassed beforehand. The idle velocities of non-degassed systems are highly variable and, therefore, not reproducible. In this case, long-term measurements are difficult. Bubbles are the result of degassing and have a very strong negative effect on the resonance system and thus on the acoustic trapping of the drops.

**Figure 8:**
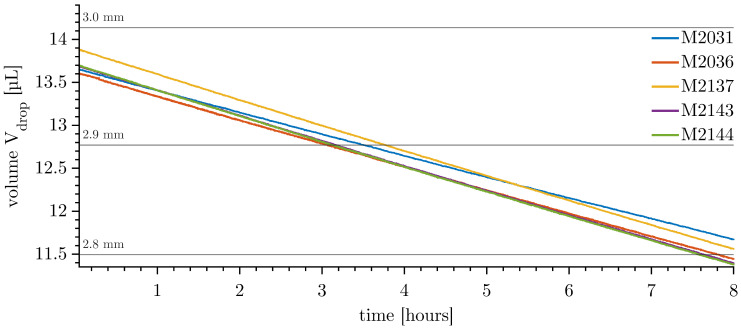
Volume of dissolving Ø 3 mm toluene drops over 8 h, with a maximum volume error of ±0.5%.

Drops of small diameter show lower reproducibility of mass transfer rates, making them the preferred choice for further investigations. Figure 8 shows the reproducibility of the dissolution of five Ø 3 mm drops in degassed ultrapure water.

The initial volume has a maximum offset of 3.5% compared to a perfect Ø 3 mm sphere, due to the random injection behaviour mentioned above. The mass transfer rates, here measured as the volume transfer rate, given as the slope of the volume trajectory, range from 0.249 to 0.293 µL/h, as shown in Table 6. The idle velocity of the measurement can be an indicator of enhanced mass transfer. Measurement M2031 has by far the smallest mass transfer rate and a small corresponding idle velocity. In addition, the temperature and, therefore, the aqueous solubility of toluene in water is reduced.

The measurements show good reproducibility if only measurements M2137, M2143, and M2144 are considered. In this case, the temperatures and the idle velocities are within a narrow range. The effect of temperature on the solubilities and, therefore, on mass transfer cannot be eliminated by tempering the system presented. An isolated tempering chamber would be required. However, the temperature is known and the effect can be quantified.

### 4.3. Flow Field in the Vicinity of the Drop

**Figure 9:**
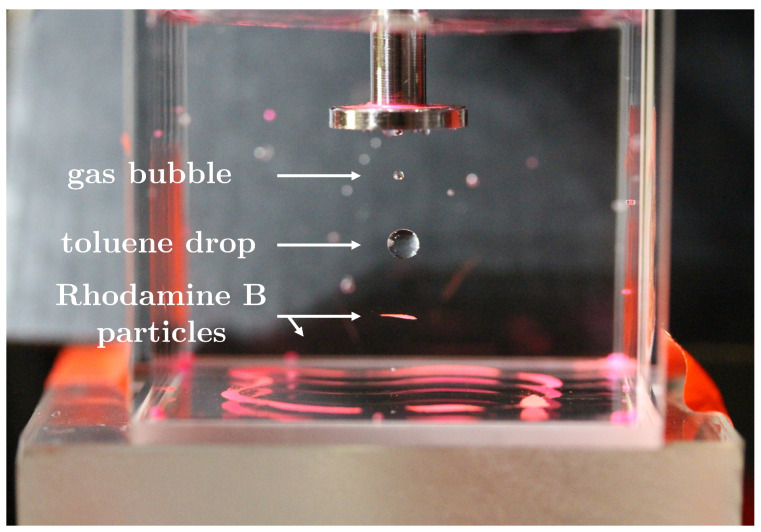
Image of the setup during PIV measurements of a toluene drop after 10 min, with Rhodamine B particles and a bubble trapped in the nodes. The ultrapure water was not initially degassed, resulting in a gas bubble.

**Particle Image Velocimetry (PIV)**: The principle of PIV is to seed a fluid with sufficient small tracer particles to visualise flow fields by tracking their paths. The main idea is that the particles have negligible sedimentation velocities and, therefore, fully follow an induced flow field. The experiments were conducted with non-degassed water accompanied by acoustic streaming, with the result shown in Figure 9.

The acoustic field collects and levitates the particles in the node, forming a circular plane. In a second node, a gas bubble was formed by releasing gas from the continuous phase caused by the ultrasonic field. Both Rhodamine B particles and gas bubbles have a positive acoustic contrast factor ϕ>0 and are attracted to the nodes in the system. The pressure field created a symmetrical particle formation at the bottom of the cuvette.

The seeded particles are not only affected by acoustic streaming but also by the standing waves themselves. Therefore, PIV cannot be used to measure the flow field in the continuous phase in this levitation system and the rainbow schlieren deflectometry technique will be used to partly visualise flow patterns in the continuous phase.

**Rainbow Schlieren Deflectometry (RSD)**: The occurring concentration gradients in the continuous phase caused by mass transfer are visualised by RSD measurements and are used for indirect flow field measurements. A prevailing flow field caused by acoustic streaming in the continuous phase would affect and deflect the uniform one-dimensional concentration streak. Two vertical directions of mass transfer, up and down, are investigated. In addition to measuring the flow field, this technique can also be used to determine the dispersion of the inhomogeneous dissolved substance.

**Figure 10:**
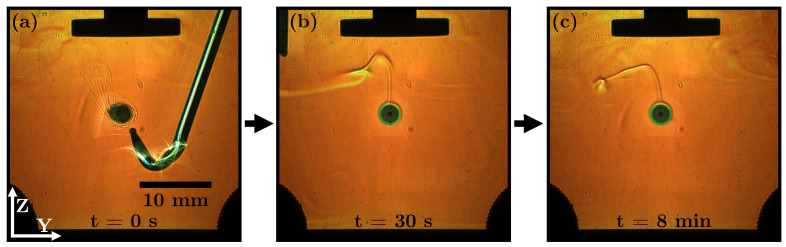
RSD images of a butyl acetate drop in water ($\rho_{d}<\rho_{c}$) of one measurement at different times: (**a**) t = 0 s, (**b**) t = 30 s and (**c**) t = 8 min.

**Butyl acetate with** $\rho_{d}<\rho_{c}$, **slightly soluble**: Figure 10a shows the injection of a butyl acetate drop; initially, a weak and disturbed concentration field is visible. Pulling out the capillary creates a turbulent flow field in the continuous phase. After about 30 s, the turbulence of the concentration field disappears (Figure 10b). From this point on, the concentration streak in the vicinity of the drop has a stationary shape, and the streak points upwards and tilts in the −y-direction. It can be assumed that no flow field is induced by acoustic streaming in close vicinity above the drop. The standing waves affect the dissolved butyl acetate and prevent it from reaching the tip of the ultrasonic transducer. It is repelled by the nodal plane halfway between the anti-node and the tip of the ultrasonic transducer.

**Figure 11:**
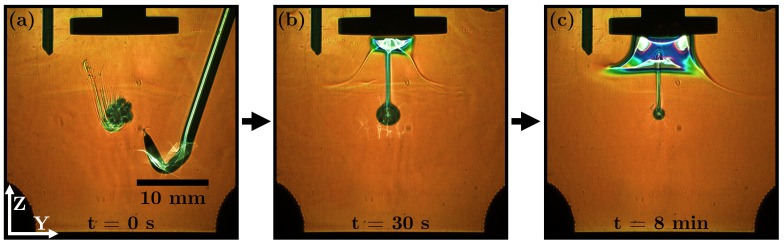
RSD images of a 1-butanol drop in water ($\rho_{d}<\rho_{c}$) of one measurement at different times: (**a**) t = 0 s, (**b**) t = 30 s and (**c**) t = 8 min.

**1-butanol with** $\rho_{d}<\rho_{c}$**, sparingly soluble**: Figure 11a shows the injection of a 1-butanol drop; initially a strong and rapidly forming concentration field is visible. The complete measurement is shown in Video S3 of the Supplementary Material. The turbulence caused by the withdrawal of the capillary has a slight and brief impact on the concentration streak. The aqueous solubility results in a rapidly dissolving drop with high mass transfer rates by observing the diameter of the drop (Figure 11b,c). The transferred and dissolved 1-butanol is trapped below the centre of the ultrasonic horn. The dissolved 1-butanol cannot pass through the volume around the edge of the plate tip (Figure 11c). In this region, the acoustic particle velocity is at its maximum and keeps 1-butanol away. It can be clearly seen that even dissolved substances can be strongly affected by standing waves.

**Figure 12:**
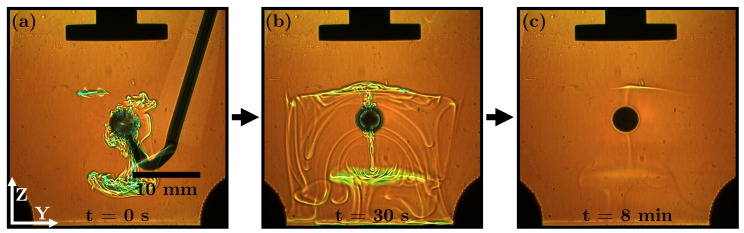
RSD images of a 5vol% propylene carbonate/toluene drop in water ($\rho_{\mathrm{TC}}>\rho_{c}$) of one measurement at different times: (**a**) t = 0 s, (**b**) t = 30 s and (**c**) t = 8 min.

**5 vol% propylene carbonate in toluene with** $\rho_{\mathrm{TC}}>\rho_{c}$**, freely soluble**: To levitate propylene carbonate (ϕ>0) in the anti-node, it is mixed with toluene, which acts as a carrier for acoustic levitation. The density of propylene carbonate is higher than that of water, so the concentration streaks in the continuous phase should theoretically only point downward. Propylene carbonate is the main transfer component (TC) in the system. The slow mass transfer of toluene is not seen in this setup, which is very slightly soluble in water. Figure 12 shows the behaviour of propylene carbonate as it is transferred to the continuous phase. The entire measurement is shown in Video S4 of the Supplementary Material. The high aqueous solubility (240 g/L) of the TC results in rapid mass transfer, leading to Marangoni eruptions driven by surface tension gradients. When the concentration of the TC in the drop falls below a threshold, the eruptions stop.

Although the density of the TC is higher than that of water, there are two opposite directions of mass transfer (Figure 12b). This behaviour is not caused by acoustic streaming; there is no superimposed turbulence visible in the spread of the TC. The acoustic field also creates two distinct barriers for the TC at two nodal planes, comparable to butyl acetate (Figure 10c) with one nodal plane as a barrier. The TC slides along these curved planes and slowly descends to the bottom of the cuvette, as it continues to dissolve. Thus, for the RSD technique, the TC visually disappears in the continuous phase. The measurement shows that the acoustic field can influence the vertical direction of mass transfer and the propagation of an inhomogeneous dissolved TC.

## 5. Conclusions

With this work, we developed a one-dimensional low-impact acoustic levitation system and investigated the application in the field of mass transfer of immobilised single drops in liquid–liquid systems in a quiescent environment.

For a systematic study, the levitation period of a long-term measurement was divided into three intervals for different behaviours: injection, short-term, and long-term. When a drop is injected, the initial movement disappears within the first 16 s. The shape is stationary after two seconds, with a circularity *C* > 0.98 regardless of the diameter; the measured volume is reliable from this point on, with an absolute volume error of ±0.5%. Short-term investigations have shown that the signal noise of the volume of a levitating drop is about ±0.05%, which is caused by the optical acquisition system. The shift of the centroid over one minute is <7.5 µm and, therefore, in the sub-pixel range, resulting in an idle velocity of $v_{i,10\mathrm{Hz}}$ < 0.5 mm/min. The covered distance has been shown to be a good visual indicator of the condition of the acoustically levitated drop. A constant slope of the covered distance, resulting in a constant idle velocity, indicates uniform and stationary levitation. The long-term measurements showed a deflection length below 7.5 µm, which is also in the sub-pixel range, ending up with an idle velocity $v_{i,10\mathrm{Hz}}$ < 0.25 mm/min. Our observations show that the acoustic field does not induce drop vibrations during operation. Measurements of dissolving drops with similar idle speeds and continuous phase temperatures are expected to have similar mass transfer rates, and these measurements have shown good reproducibility. The importance of degassing the liquid phases has been addressed to perform reproducible measurements with this technique. When gas is dissolved in the dispersed and in the continuous phase, significant vibrations can be observed with an idle velocity $v_{i,10\mathrm{Hz}}$ > 15 mm/min, resulting in enhanced mass transfer.

Rainbow schlieren deflectometry measurements have shown that the standing waves do not induce a flow field caused by acoustic streaming in the vicinity of the drop. Any compressibility in the liquid environment will result in an acoustic streaming flow, such as gas bubbles, submerged materials (e.g., PTFE), or dissolved gases in the liquid phases. However, the dispersion of inhomogeneous dissolved substances in the continuous phase was significantly affected by the standing waves in two different ways. One group of substances is prevented from spreading through the nodal planes and is additionally pulled towards these planes against its natural buoyancy. The inhomogeneous dissolved substance shows an alternative acoustic contrast factor compared to the pure substance in water. The other group of substances can pass through the nodal planes without interference and is stopped by regions of maximum particle velocity of the acoustic field. In this case, the substance is trapped under the horn of the ultrasonic transducer and shows a negative contrast factor as an inhomogeneous dissolved substance. These observations require further investigations to understand this mechanism in more detail.

Acoustic levitation can be a unique tool for many directions of fundamental research in liquid–liquid systems, such as mass transfer measurements, medical ageing tests without contact with any surfaces, and micro-reactors with excellent tempering conditions, to mention a few. To perform measurements in a system with continuous phases other than water, it is essential to adapt the design of the acoustic levitation cell and its resonance behaviour to the specific physical properties of the chosen substance.

## Figures and Tables

**Table 1:** Specifications of the developed acoustic levitation system.

	Variation Range
contact time	seconds to days
initial drop diameter (volume)	Ø 2–5.5 mm (4.19–87.11 µL)
ambient temperature	18–24 ${}^{\circ }$C
dispersed phase	solvents (ϕ<0)
continuous phase	ultrapure water

**Table 2:** Physical properties of the used pure substances and the mixture at 20 ${}^{\circ }$C.

				Aqueous		Speed	Acoustic Contrast
		**Manufacturer**	**Purity**	**Solubility**	Density *	of Sound *	**Factor** ϕ * (Equation (1**))**
			**[wt%]**	**[g/L]**	**[g/mL]**	**[m/s]**	**[-]**
dispersed phase	toluene	Supelco	≥99.9	0.5 ${}^{+}$	0.86691	1320.34	−0.199
	butyl acetate	Supelco	≥99.5	6.8 ${}^{+}$	0.88147	1148.77	−0.338
	1-butanol	ChemSolute	≥99.5	84 ${}^{+}$	0.80968	1256.41	−0.311
	propylene carbonate	Carl Roth	≥99.7	240 ${}^{m}$	1.20466	1461.75	0.110
5vol% propylene carbonate in toluene		-	-	-	0.88462	1150.94	−0.332
continuous phase	ultrapure water				0.99829	1482.46	0

* Own measurements with an Anton Paar Density and Sound Velocity Meter DSA 5000 M. ${}^{+}$ Data from [24], if needed, are interpolated with a polynomial of the fourth degree to 20 ${}^{\circ }$C. ${}^{m}$ Given in manufacturer datasheet.

**Table 3:** Determination of the optical accuracy with calibration targets over 180 s.

Calibration Target		Optically Acquired		Difference				Idle Velocity
**Diameter**	**Volume**	**Diameter**	**Volume**	Δ **Diameter**		Δ **Volume**		$v_{i,10\mathrm{Hz}}$
**[mm]**	**[µL]**	**[mm]**	**[µL]**	**[mm]**	**[%]**	**[µL]**	**[%]**	**[mm/min]**
1.00	0.5236	0.9984 ± 3.34 $\times \;10^{-4}$	0.5211 ± 5.23 $\times \;10^{-4}$ $\hat{=}$ 2.01‰	0.0016	0.1581	0.0025	0.4735	0.0562
5.00	65.4499	4.9960 ± 2.31 $\times \;10^{-4}$	65.2921 ± 90.61 $\times \;10^{-4}$ $\hat{=}$ 0.28‰	0.0040	0.0804	0.1578	0.2411	0.0244

**Table 4:** Short-term characteristics over 60 s of representative toluene drops.

Target Diameter	Diameter	Volume	Deflection Length	Circularity	Elliptical Shape Parameters as in Figure 2c			Idle Velocity
d	$d_{s}$	$V_{\mathrm{drop}}$	$l_{d,\mathrm{yz}}$	C	$a_{e}$	$b_{e}$	$\gamma_{e}$	$v_{i,10\mathrm{Hz}}$
**[mm]**	**[mm**±**‰]**	**[µL**±**‰]**	**[µm]**	**[-**±**‰]**	**[mm** ± **µm]**	**[mm** ± **µm]**	[°]	**[$\frac{\mathrm{mm}}{\min}$]**
3.0	2.98 ± 0.15	13.79 ± 0.44	0.62 ± 1.85	1.000 ± 3.98	2.99 ± 0.57	2.96 ± 0.56	−1.23 ± 0.77	0.49
3.5	3.47 ± 0.12	21.82 ± 0.35	0.40 ± 0.69	0.997 ± 5.89	3.49 ± 0.66	3.43 ± 0.84	−1.12 ± 0.33	0.24
4.0	4.01 ± 0.10	33.85 ± 0.31	0.54 ± 1.27	0.996 ± 8.37	4.05 ± 0.71	3.95 ± 0.59	−1.10 ± 0.20	0.24
4.5	4.48 ± 0.13	47.04 ± 0.39	0.76 ± 1.99	0.995 ± 7.24	4.54 ± 0.80	4.37 ± 1.24	−1.24 ± 0.17	0.54
5.0	4.98 ± 0.09	64.56 ± 0.27	0.49 ± 1.18	0.992 ± 5.41	5.04 ± 0.73	4.85 ± 0.59	−0.51 ± 0.10	0.34
5.5	5.49 ± 0.11	86.62 ± 0.34	0.46 ± 0.67	0.990 ± 5.76	5.58 ± 0.79	5.31 ± 0.60	−0.61 ± 0.10	0.33

The units of the values are structured as follows: mean ± (max − min)/2 [unit] or mean ± (500·(max − min)/mean) [unit ± ‰].

**Table 5:** Idle velocity of toluene drops in degassed (Figure 6) and non-degassed (Figure 7) systems, 8 h long-term behaviour.

Diameter	Idle Velocity	
d	$v_{i,10\mathrm{Hz},\mathrm{degassed}}$	$v_{i,10\mathrm{Hz},\mathrm{non}-\mathrm{degassed}}$
**[mm]**	**[mm/min]**	**[mm/min]**
3	0.2415	1.509
4	0.1855	16.544
5	0.2255	8.4570

**Table 6:** Characteristic measures of the measurements shown in Figure 8.

			M2031	M2036	M2137	M2143	M2144
mass transfer rate	${\dot{V}}_{d}$	[µL/h]	0.249	0.272	0.293	0.290	0.293
idle velocity	$v_{i,10Hz}$	[mm/min]	0.15	0.24	0.22	0.20	0.24
temperature	${\bar{T}}_{c}$	[${}^{\circ }$C]	18.0	19.3	22.9	23.0	23.1

## Data Availability

The data that support the findings of this study are available on request.

## Supplementary Materials

The following supporting information can be downloaded at: https://www.mdpi.com/article/10.3390/mi14101923/s1. Video S1: Injection of a toluene drop (20 s, HSC), Video S2: Long-term measurement of a toluene drop (8 h, HSC), Video S3: 1-butanol drop (8 min, RSD) and Video S4: 5vol% propylene carbonate in toluene drop (8 min, RSD).

## Abbreviations

    The following abbreviations are used in this manuscript:

FPGA	Field programmable gate array
HSC	High-speed camera
CMA	Centred moving average
PTFE	Polytetrafluoroethylene
PIV	Particle image velocimetry
RSD	Rainbow schlieren deflectometry
TC	Transfer component
